# IMPACTO-MR: a Brazilian nationwide platform study to assess infections and multidrug resistance in intensive care units

**DOI:** 10.5935/0103-507X.20220209-en

**Published:** 2022

**Authors:** Bruno M Tomazini, Antonio Paulo Nassar Jr, Thiago Costa Lisboa, Luciano César Pontes de Azevedo, Viviane Cordeiro Veiga, Daniela Ghidetti Mangas Catarino, Debora Vacaro Fogazzi, Beatriz Arns, Filipe Teixeira Piastrelli, Camila Dietrich, Karina Leal Negrelli, Isabella de Andrade Jesuíno, Luiz Fernando Lima Reis, Renata Rodrigues de Mattos, Carla Cristina Gomes Pinheiro, Mariane Nascimento Luz, Clayse Carla da Silva Spadoni, Elisângela Emilene Moro, Flávia Regina Bueno, Camila Santana Justo Cintra Sampaio, Débora Patrício Silva, Franca Pellison Baldassare, Ana Cecilia Alcantara Silva, Thabata Veiga, Leticia Barbante, Marianne Lambauer, Viviane Bezerra Campos, Elton Santos, Renato Hideo Nakawaga Santos, Ligia Nasi Laranjeiras, Nanci Valeis, Eliana Santucci, Tamiris Abait Miranda, Ana Cristina Lagoeiro do Patrocínio, Andréa de Carvalho, Eduvirgens Maria Couto de Sousa, Ancelmo Honorato Ferraz de Sousa, Daniel Tavares Malheiro, Isabella Lott Bezerra, Mirian Batista Rodrigues, Julliana Chicuta Malicia, Sabrina Souza da Silva, Bruna dos Passos Gimenes, Guilhermo Prates Sesin, Alexandre Prehn Zavascki, Daniel Sganzerla, Gregory Saraiva Medeiros, Rosa da Rosa Minho dos Santos, Fernanda Kelly Romeiro Silva, Maysa Yukari Cheno, Carolinne Ferreira Abrahão, Haliton Alves de Oliveira Junior, Leonardo Lima Rocha, Pedro Aniceto Nunes Neto, Valéria Chagas Pereira, Luis Eduardo Miranda Paciência, Elaine Silva Bueno, Eliana Bernadete Caser, Larissa Zuqui Ribeiro, Caio Cesar Ferreira Fernandes, Juliana Mazzei Garcia, Vanildes de Fátima Fernandes Silva, Alisson Junior dos Santos, Flávia Ribeiro Machado, Maria Aparecida de Souza, Bianca Ramos Ferronato, Hugo Corrêa de Andrade Urbano, Danielle Conceição Aparecida Moreira, Vicente Cés de Souza-Dantas, Diego Meireles Duarte, Juliana Coelho, Rodrigo Cruvinel Figueiredo, Fernanda Foreque, Thiago Gomes Romano, Daniel Cubos, Vladimir Miguel Spirale, Roberta Schiavon Nogueira, Israel Silva Maia, Cassio Luis Zandonai, Wilson José Lovato, Rodrigo Barbosa Cerantola, Tatiana Gozzi Pancev Toledo, Pablo Oscar Tomba, Joyce Ramos de Almeida, Luciana Coelho Sanches, Leticia Pierini, Mariana Cunha, Michelle Tereza Sousa, Bruna Azevedo, Felipe Dal-Pizzol, Danusa de Castro Damasio, Marina Peres Bainy, Dagoberta Alves Vieira Beduhn, Joana D’Arc Vila Nova Jatobá, Maria Tereza Farias de Moura, Leila Rezegue de Moraes Rego, Adria Vanessa da Silva, Luana Pontes Oliveira, Eliene Sá Sodré Filho, Silvana Soares dos Santos, Itallo de Lima Neves, Vanessa Cristina de Aquino Leão, João Lucidio Lobato Paes, Marielle Cristina Mendes Silva, Cláudio Dornas de Oliveira, Raquel Caldeira Brant Santiago, Jorge Luiz da Rocha Paranhos, Iany Grinezia da Silva Wiermann, Durval Ferreira Fonseca Pedroso, Priscilla Yoshiko Sawada, Rejane Martins Prestes, Glícia Cardoso Nascimento, Cintia Magalhães Carvalho Grion, Claudia Maria Dantas de Maio Carrilho, Roberta Lacerda Almeida de Miranda Dantas, Eliane Pereira Silva, Antônio Carlos da Silva, Sheila Mara Bezerra de Oliveira, Nicole Alberti Golin, Rogerio Tregnago, Valéria Paes Lima, Kamilla Grasielle Nunes da Silva, Emerson Boschi, Viviane Buffon, André Sant’Ana Machado, Leticia Capeletti, Rafael Botelho Foernges, Andréia Schubert de Carvalho, Lúcio Couto de Oliveira Junior, Daniela Cunha de Oliveira, Everton Macêdo Silva, Julival Ribeiro, Francielle Constantino Pereira, Fernanda Borges Salgado, Caroline Deutschendorf, Cristofer Farias da Silva, Andre Luiz Nunes Gobatto, Carolaine Bomfim de Oliveira, Marianna Deway Andrade Dracoulakis, Natália Oliveira Santos Alvaia, Roberta Machado de Souza, Larissa Liz Cardoso de Araújo, Rodrigo Morel Vieira de Melo, Luiz Carlos Santana Passos, Claudia Fernanda de Lacerda Vidal, Fernanda Lopes de Albuquerque Rodrigues, Pedro Kurtz, Cássia Righy Shinotsuka, Maria Brandão Tavares, Igor das Virgens Santana, Luciana Macedo da Silva Gavinho, Alaís Brito Nascimento, Adriano J Pereira, Alexandre Biasi Cavalcanti

**Affiliations:** 1 Hospital Sírio-Libanês - São Paulo (SP), Brazil.; 2 Brazilian Research in Intensive Care Network (BRICNet) - São Paulo (SP), Brazil.; 3 Hospital Israelita Albert Einstein - São Paulo (SP), Brazil.; 4 Hospital A. C. Camargo Cancer Center - São Paulo (SP), Brazil.; 5 Research Institute, HCor-Hospital do Coração - São Paulo (SP), Brazil.; 6 BP - A Beneficência Portuguesa de São Paulo - São Paulo (SP), Brazil.; 7 Hospital Alemão Oswaldo Cruz - São Paulo (SP), Brazil.; 8 Hospital Moinhos de Vento - Porto Alegre (RS), Brazil.; 9 Hospital Federal de Ipanema - Rio de Janeiro (RJ), Brazil.; 10 Hospital Unimed Limeira - Limeira (SP), Brazil.; 11 Hospital Unimed Vitória - Vitória (ES), Brazil.; 12 Hospital Estadual Mário Covas - Santo André (SP), Brazil.; 13 Santa Casa de Misericórdia de Passos - Passos (MG), Brazil.; 14 Hospital São Paulo, Escola Paulista de Medicina, Universidade Federal de São Paulo - São Paulo (SP), Brazil.; 15 Hospital Erasto Gaertner - Curitiba (PR), Brazil.; 16 Hospital Vila da Serra - Nova Lima (MG), Brazil.; 17 Hospital Universitário Clementino Fraga Filho, Universidade Federal do Rio de Janeiro - Rio de Janeiro (RJ), Brazil.; 18 Hospital Maternidade São José - Colantina (ES), Brazil.; 19 Hospital e Maternidade São Luiz Itaim - São Paulo (SP), Brazil.; 20 Hospital Aviccena - São Paulo (SP), Brazil.; 21 Hospital Nereu Ramos - Florianópolis (SC), Brazil.; 22 Hospital das Clínicas, Faculdade de Medicina de Ribeirão Preto, Universidade de São Paulo - Ribeirão Preto (SP), Brazil.; 23 Hospital e Maternidade Brasil - Santo André (SP), Brazil.; 24 Hospital de Amor Jales - Jales (SP), Brazil.; 25 Hospital de Amor - Barretos (SP), Brazil.; 26 Fundação Hospitalar São Francisco de Assis - Belo Horizonte (MG), Brazil.; 27 Hospital São José - Criciúma (SC), Brazil.; 28 Hospital Escola, Universidade Federal de Pelotas - Pelotas (RS), Brazil.; 29 Hospital do Tricentenário - Olinda (PE), Brazil.; 30 Hospital Jean Bitar - Belém (PA), Brazil.; 31 Hospital Presidente Vargas, São Luís (MA), Brazil.; 32 Hospital Estadual de Aparecida de Goiânia Cairo Louzada - Goiânia (GO), Brazil.; 33 Hospital Regional Público do Leste do Pará - Paragominas (PA), Brazil.; 34 Santa Casa de Misericórdia de Belo Horizonte - Belo Horizonte (MG), Brazil.; 35 Santa Casa de Misericórdia de São João Del Rei - São João Del Rei (MG), Brazil.; 36 Hospital Estadual Alberto Rassi - Goiânia (GO), Brazil.; 37 Hospital Universitário, Universidade Federal do Piauí -Teresina (PI), Brazil.; 38 Hospital Universitário, Universidade Estadual de Londrina - Londrina (PR), Brazil.; 39 Hospital Universitário Onofre Lopes, Universidade Federal do Rio Grande do Norte - Natal (RN), Brazil.; 40 Hospital Regional do Baixo Amazonas - Santarém (PA), Brazil.; 41 Hospital Tacchini - Bento Gonçalves (RS), Brazil.; 42 Hospital Universitário de Brasília, Universidade de Brasília - Brasília (DF), Brazil.; 43 Hospital Geral de Caxias do Sul - Caxias do Sul (RS), Brazil.; 44 Hospital Ernesto Dornelles - Porto Alegre (RS), Brazil.; 45 Hospital Santa Cruz - Santa Cruz (RS), Brazil.; 46 Hospital Geral Cleriston de Andrade - Feira de Santana (BA), Brazil.; 47 Hospital Base do Distrito Federal - Brasília (DF), Brazil.; 48 Hospital Municipal de Maringá. - Maringá (PR), Brazil.; 49 Hospital de Clínicas de Porto Alegre, Universidade Federal do Rio Grande do Sul - Porto Alegre (RS), Brazil.; 50 Hospital da Cidade - Salvador (BA), Brazil.; 51 Hospital da Bahia - Salvador (BA), Brazil.; 52 Hospital São Lucas - Aracajú (SE), Brazil.; 53 Hospital Ana Nery - Salvador (BA), Brazil.; 54 Hospital das Clínicas, Universidade Federal de Pernambuco - Recife (PE), Brazil.; 55 Instituto Estadual do Cérebro Paulo Niemeyer - Rio de Janeiro (RJ), Brazil.; 56 Hospital do Subúrbio - Salvador (BA), Brazil.; 57 Fundação Hospital de Clínicas Gaspar Vianna - Belém (PA), Brazil.

**Keywords:** Database, Database management systems, Software, IMPACTO-MR, Bacterial infections, Drug-resistance, bacterial, Intensive care units

## Abstract

**Objective:**

To describe the IMPACTO-MR, a Brazilian nationwide intensive care unit platform study focused on the impact of health care-associated infections due to multidrug-resistant bacteria.

**Methods:**

We described the IMPACTO-MR platform, its development, criteria for intensive care unit selection, characterization of core data collection, objectives, and future research projects to be held within the platform.

**Results:**

The core data were collected using the Epimed Monitor System® and consisted of demographic data, comorbidity data, functional status, clinical scores, admission diagnosis and secondary diagnoses, laboratory, clinical, and microbiological data, and organ support during intensive care unit stay, among others. From October 2019 to December 2020, 33,983 patients from 51 intensive care units were included in the core database.

**Conclusion:**

The IMPACTO-MR platform is a nationwide Brazilian intensive care unit clinical database focused on researching the impact of health care-associated infections due to multidrug-resistant bacteria. This platform provides data for individual intensive care unit development and research and multicenter observational and prospective trials.

## INTRODUCTION

In Critical Care Medicine, high-quality clinical databases are a major breakthrough now recognized as an integral part of critical care practice, research, benchmarking, and performance evaluation.^(1,2)^ Known examples are the Australian and New Zealand Intensive Care Society (ANZICS),^(1)^ The Intensive Care National Audit & Research Center (ICNARC) in the United Kingdom,^(3)^ the National Intensive Care Evaluation (NICE),^(4)^ and the Medical Information Mart for Intensive Care III (MIMIC III) in the United States.^(5)^

From a research standpoint, a multicentric clinical database of intensive care units (ICUs) that takes into account regional and economic heterogeneities and provides prospective capture of a large amount of data from individual patients creates new perspectives for observational and epidemiological research,^(1-4)^ and can be the backbone for both platforms and other clinical trials. This representativeness aspect is markedly important in low- and middle-income countries, such as Brazil, where within-country disparities clearly impact the care process and patient outcomes.^(6,7)^ Not acknowledging these differences might undermine the external validity of both epidemiological and randomized clinical trials.^(8,9)^

Given the epidemic of antimicrobial resistance worldwide,^(10-12)^ which is especially relevant in ICUs, where the frequency of health care-associated infections (HAIs) and antimicrobial utilization are higher,^(13,14)^ coupled with higher densities of HAIs in developing countries,^(15)^ we have a suitable and rich scenario for data generation and future clinical trials.

This manuscript describes the development and characterization of the Impact of Infections by Antimicrobial-Resistant Microorganisms in Patients Admitted to Adult Intensive Care Units in Brazil: Platform of Projects to Support the National Action Plan for the Prevention and Control of Antimicrobial Resistance (IMPACTO-MR), a Brazilian nationwide ICU platform study focused on the impact of HAIs due to multidrug-resistant (MDR) bacteria.

## METHODS

### Development

The IMPACTO-MR program is developed and coordinated in a partnership between the hospitals members of the Program to Support Institutional Development of Universal Health System (*Programa de Apoio ao Desenvolvimento Institucional do Sistema Único de Saúde* - PROADI-SUS): *Hospital Alemão Oswaldo Cruz* (HAOC), *Hospital Israelita Albert Einstein* (HIAE), *Hospital Moinhos de Vento* (HMV), *Hospital Sírio-Libanês* (HSL), and *HCor-Hospital do Coração* (IP-HCor) in a collaboration with the Brazilian Research in Intensive Care Network (BRICNet) and is supported and overseen by the Department of Science and Technology from the Brazilian Ministry of Health (DECIT/SCTIE/MS) and by the General Management of Health Technologies of the Brazilian Health Regulatory Agency (*Gerência Geral de Tecnologias em Saúde da Agência Nacional de Vigilância Sanitária* - GGTES/ANVISA). In 2022, *BP - A Beneficência Portuguesa de São Paulo* joined the other hospitals in coordination with the project. The project is funded by the PROADI-SUS, a nationwide program aimed at strengthening and qualifying the Brazilian Universal Health System (SUS) throughout the country.

The program is developed as a prospective, multicentric platform study where participating ICUs would collect data on all admitted adult patients (≥ 18 years old) on a specific data capture system that constitutes the study’s core database. This core database would initially provide data to prospective observational studies within the platform, and each database might have specifically designed additional databases as needed. Additionally, this platform would provide data for future randomized embedded controlled trials (as registry-based clinical trials and/or adaptive designs).

Discussion on the platform and database design began in late 2018. The study’s protocol was approved by the coordinator site’s Institutional Review Board (IRB) in November 2018 (approval number 3,025,217). In addition, before each participant site startup, the protocol was approved by their IRB. All but one institution waived the need for informed consent for patient data capture. Patient inclusion began in October 2019 and is expected to continue until December 2023.

### Intensive care unit selection

Each hospital had to fulfill all the following eligibility criteria to participate in the study:

- Have an Infection Prevention and Control Committee.
- Perform monthly notifications of HAIs and MDR to the Health Care-associated Infections National Epidemiological Surveillance System.
- Have an ICU with at least six beds.
- Have a microbiology laboratory.
- Utilize or be willing to utilize one of the following antimicrobial susceptibility testing criteria: Brazilian Committee on Antimicrobial Susceptibility Testing (BrCAST),^(16)^ European Committee on Antimicrobial Susceptibility Testing (EUCAST)^(17)^ or Clinical and Laboratory Standards Institute (CLSI).^(18)^

The aim was to include at least 50 ICUs nationwide and to account for the geographical and socioeconomic heterogeneity of Brazil, so some proportions were to be followed. First, the proportion of 70% of public or philanthropic hospitals and 30% of private hospitals, and second, the number of ICUs included in each Brazilian geographic region (North, Northeast, Central-West, Southeast, and South) should be proportional to the availability of ICU beds in each region; therefore, more populated areas, such as South and Southeast, would have more ICUs.

**Figure 1:**
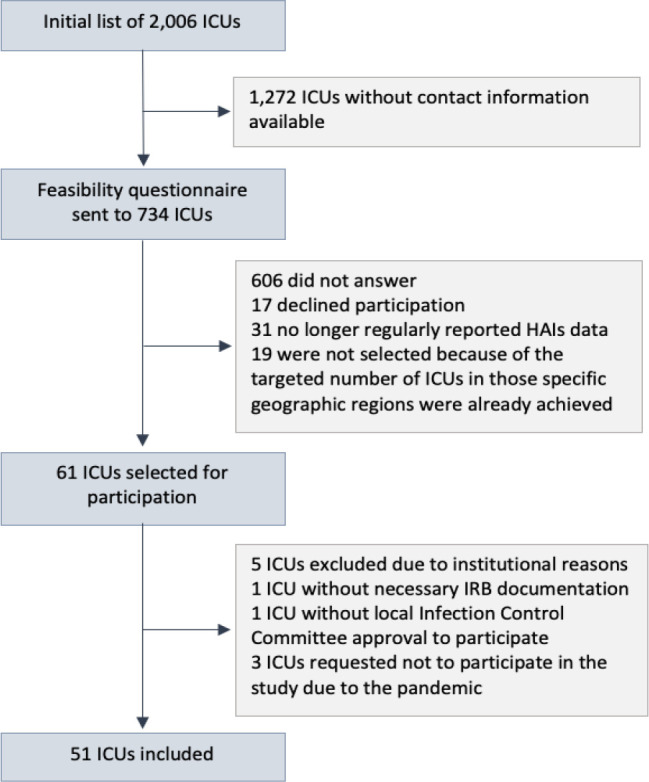
Study flowchart.

**Figure 2:**
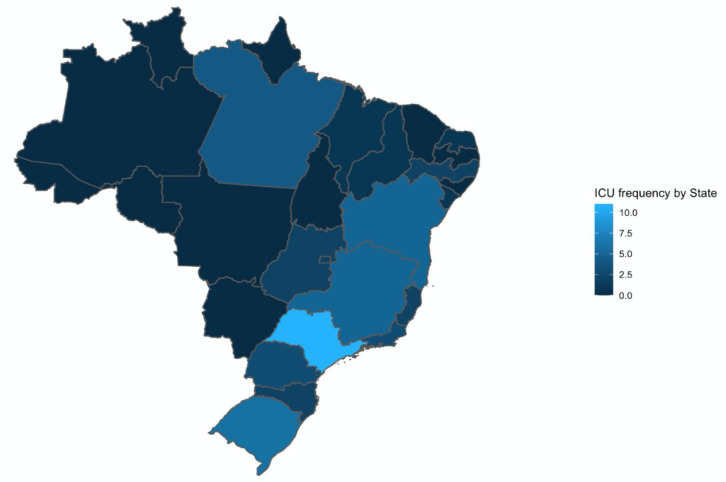
Geographical distribution of participating intensive care units.

From a provided list of 2,000 ICUs (that had regularly reported HAIs data to the ANVISA in 2016), we sent a feasibility questionnaire to 728 ICUs from which we had contact information available. Given the need to have 10 hospitals with a minimum infrastructure of costs and the ability to provide such data on a patient-level basis (for the costs’ substudy), a second look into the abovementioned list (covering all hospitals) was performed to complete the selection. The criteria for the cost substudy were as follows: (1) local use of a computerized cost system; (2) local accounting system using different cost centers per area; and (3) material and medications controlled at the patient level (without any type of apportionment). Six additional hospitals indicated by the ANVISA and Ministry of Health were also considered for the cost substudy and received the invitation. The platform design allowed for ICU exclusions and inclusions during the study, with the aim of maintaining approximately 50 ICUs participating. Six hundred fifty-four ICUs did not meet the inclusion criteria or were unwilling to participate in the study, and 19 ICUs were not selected because the number of participating ICUs in their geographic region was already achieved. Of the 61 initially selected ICUs, 51 were included in the study (Figures 1 and 2).

ICU - intensive care unit; HAIs - health care-associated infections; IRB - Institutional Review Board.

ICU - intensive care unit

### Data collection

Data were collected using the Epimed Monitor System® (Epimed Solutions®, Rio de Janeiro, Brazil), a secured commercial cloud-based registry for quality improvement and benchmarking purposes,^(2)^ customized for the study’s objectives. The software was provided to all participating centers. We collected demographic data, comorbidity data (using the Charlson Comorbidity Index),^(19)^ functional status (adapted from the Eastern Cooperative Oncology Group - ECOG),^(20)^ Simplified Acute Physiology Score III (SAPS 3),^(21)^ Sequential Organ Failure Assessment (SOFA) score,^(22)^ admission type (medical, elective surgery or emergency/urgent surgery), admission diagnosis and secondary diagnoses, laboratory, clinical, and microbiological data, and organ support during ICU stay, among others. The core individual patient data collected are displayed in table 1.

**Table 1:** Core individual patient data collected

Demographic data	Baseline data (at ICU admission)	Daily data (during ICU stay)	Microbiological data (during ICU stay)	At ICU discharge	At hospital discharge
Gender	Hospital admission date	Antibiotic use	Microbiological culture results^*^	Discharge date	Discharge date
Age	ICU admission date	Infection type		Health status	Health status
Weight	Main diagnosis and admission type	Detailed diagnostic criteria if ventilator-associated pneumonia, catheter-associated urinary tract infections, and catheter-related bloodstream infection			
Height	Comorbidities and functional status	Use of mechanical ventilation, urinary catheter, and central venous catheter			
Zip code	Origin before admission				
	SAPS 3 and SOFA Score variables				
	Complications				
	Antibiotic use in the past 30 days				
	Presence of infection				
	Laboratory dat†				
	Vital signs‡				
	Use of support therapies§				

ICU - intensive care unit; SAPS 3 - Simplified Acute Physiology Score 3; SOFA - Sequential Organ Failure Assessment.

* Data on microorganisms and antibiotic resistance of all microbiological cultures collected in the intensive care unit; † creatinine, platelet count, leukocytes, urea bilirubin, lactate, pH, PaO_2_, PaCO_2_; ‡ heart rate, respiratory rate, diastolic blood pressure, systolic blood pressure; § vasopressor use, mechanical ventilation.

Data input was performed through a structured electronic case report form (eCRF) by manual entry or, in some cases, through integration with the hospital’s electronic records. Patient data are entered into the eCRF prospectively, except on weekend and holiday admissions (for some ICUs), and pass through an automated anonymization process within the Epimed System. Unique identifiers were generated for each patient included in the database and each participating ICU.

Regarding costs, patient-level fixed and variable costs were calculated monthly and informed (5 hospitals, one of each region) or quarterly (the other 5) and validated by a team of specialists in the field. A proprietary system (“e-Custos IMPACTO MR”, São Paulo/Brazil) was developed to consolidate patient-level and item-level data and integrate it with Epimed data (by an Application Programming Interface - API).

Clinical data quality control and data management were centralized with the data management team of HCor Research Institute, which generated biweekly data quality reports sent to each site. Additionally, the Epimed System provides automatic interactive assessment of the data. Each participating institution designated data collectors who were trained by the IMPACTO-MR team and by Epimed Solutions®. Additionally, the study organization provided operational manuals and telephone support to each participating center. Regarding cost data, a specific Data Management Plan was created, and HIAE was responsible for its execution.

An *in-loco* initiation visit was planned for each participating center; however, due to travel restrictions in Brazil during the COVID-19 pandemic, some centers were initiated after an online visit.

### Privacy and confidentiality

Data are stored initially in the Epimed cloud system, according to the international security protocol. These data were automatically anonymized before being sent to the study’s data management team. Only the study committee and data management team have access to these data. In the same way, “e-Custos” handles only anonymized data and has restricted access controlled by different profiles authenticated by unique login/passwords.

### Data ownership

Each contributing ICU shares ownership of its submitted data with the study committee and the Brazilian Ministry of Health. Patient deidentified data might be available to research teams from the participating institutions upon approval by the study committee and the Brazilian Ministry of Health.

### Data records

**Figure 3:**
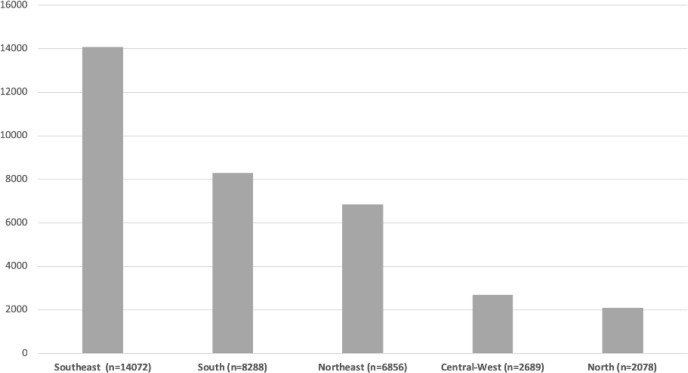
Proportion of patients included in each region.

From October 2019 to December 2020, 33,983 patients from 51 ICUs were included in the core database (Table 2). The proportion of patients included in each Brazilian region is shown in figure 3. Data capture is ongoing in 40 centers, with more than 70,000 patients included as of February 2022.

**Table 2:** List of all participating intensive care units

Hospital name	State	City	Geographic region
*Hospital Ernesto Dornelles*	RS	Porto Alegre	South
*Hospital Aviccena*	SP	São Paulo	Southeast
*Hospital São José - Criciúma*	SC	Criciúma	South
*Hospital e Maternidade Brasil (Rede D’Or São Luis)*	SP	Santo André	Southeast
*Hospital Vila da Serra (Instituto Materno Infantil de Minas Gerais S/A)*	MG	Nova Lima	Southeast
*Hospital de Clínicas de Porto Alegre*	RS	Porto Alegre	South
*Santa Casa de Misericórdia de Passos*	MG	Passos	Southeast
*Hospital Tacchini*	RS	Bento Gonçalves	South
*Hospital da Bahia (HBA S/A Assistência Médica e Hospitalar)*	BA	Salvador	Northeast
*Santa Casa de Belo Horizonte*	MG	Belo Horizonte	Southeast
*Hospital Regional do Baixo Amazonas do Pará*	PA	Santarém	North
*Hospital do Subúrbio*	BA	Salvador	Northeast
*BP - A Beneficência Portuguesa de São Paulo*	SP	São Paulo	Southeast
*Hospital Maternidade São José - Fundação Social Rural de Colatina*	ES	Colatina	Southeast
*Hospital Universitário Onofre Lopes*	RN	Natal	Northeast
*Hospital Estadual Geral de Goiânia*	GO	Goiânia	Midwest
*Hospital Ana Nery*	BA	Salvador	Northeast
*Hospital São Luiz Itaim*	SP	São Paulo	Southeast
*Hospital Santa Cruz*	RS	Santa Cruz do Sul	South
*A.C. Camargo Cancer Center*	SP	São Paulo	Southeast
*Hospital Universitário da Universidade Federal do Piauí*	PI	Teresina	Northeast
*Hospital Universitário de Brasília*	DF	Brasília	Midwest
*Hospital da Cidade*	BA	Salvador	Northeast
*Hospital Universitário Clementino Fraga Filho*	RJ	Rio de Janeiro	Southeast
*Instituto Estadual do Cérebro Paulo Niemeyer*	RJ	Rio de Janeiro	Southeast
*Hospital Regional Público do Leste do Pará*	PA	Paragominas	North
*Instituto Hospital de Base (Instituto de Gestão Estratégica de Saúde do Distrito Federal)*	DF	Brasília	Midwest
*Hospital Geral de Caxias do Sul*	RS	Caxias do Sul	South
*Hospital Federal de Ipanema*	RJ	Rio de Janeiro	Southeast
*Hospital São Lucas*	SE	Aracaju	Northeast
*HCor-Hospital do Coração*	SP	São Paulo	Southeast
*UNIMED Vitória*	ES	Vitória	Southeast
*Hospital Municipal de Maringá (Fundo Municipal de Saúde)*	PR	Maringá	South
*Hospital Tricentenário*	PE	Recife	Northeast
*Hospital das Clínicas da Faculdade de Medicina de Ribeirão Preto da Universidade de São Paulo*	SP	Ribeirão Preto	Southeast
*Hospital Estadual de Urgências de Aparecida de Goiânia*	GO	Aparecida de Goiânia	Midwest
*Santa Casa de Misericórdia de São João del Rei*	MG	São João Del Rei	Southeast
*Hospital de Amor (Fundação PIO XII)*	SP	Barretos	Southeast
*Hospital Erasto Gaertner*	PR	Curitiba	South
*Hospital Unimed Limeira*	SP	Limeira	Southeast
*Hospital Estadual Mário Covas*	SP	Santo André	Southeast
*Hospital Escola da Universidade Federal de Pelotas*	RS	Pelotas	South
*Fundação Hospital de Clínicas Gaspar Viana*	PA	Belém	North
*Hospital Jean Bitar*	PA	Belém	North
*Hospital do Câncer de Barretos - Unidade III Jales*	SP	Barretos	Southeast
*Hospital da Universidade Estadual de Londrina*	PR	Londrina	South
*Hospital Nereu Ramos*	SC	Florianópolis	South
*Hospital Presidente Vargas*	MA	São Luís	Northeast
*Fundação São Francisco de Assis*	MG	Belo Horizonte	Southeast
*Hospital Geral Cleriston de Andrade*	BA	Feira de Santana	Northeast
*Hospital das Clínicas da Universidade Federal de Pernambuco*	PE	Recife	Northeast

### Research projects within the platform

Initially, the platform subsided core data to five prospective observational projects aimed at evaluating different aspects of the MDR dynamics and its consequences. Briefly, these projects studied the following aspects:

- Evaluation of the Infection Control Committees and microbiology labs within each participating institution.
- Evaluation of the clinical impact of MDR acquisition.
- Evaluation of the economic impact of MDR.
- Evaluation of risk factors for acquisition of MDR.
- Comparison of reported and notified data on HAIs.

Proposals for observational studies and secondary analyses using the database can be submitted by each participating site center and are individually evaluated by their scientific merits by the study committee. Additionally, beginning in early 2022, the platform will provide data to two observational trials and four prospective randomized trials, including two trials on antibiotic duration for specific HAIs and two cluster randomized trials on interventions to decrease MDR incidence.

## DISCUSSION

This manuscript describes the development and core structure of the IMPACTO-MR platform, a multicenter database of Brazilian ICUs, and a pioneering initiative in Latin America that is providing real world data allowing for focused research in HAIs.

Successful databases share common characteristics: a multidisciplinary team, stable funding, focused goals, data collection, focused design, and relevant leadership.^(23)^

In a continental country such as Brazil, having a comprehensive and representative clinical database is a monumental task. Regional socioeconomic disparities and resource availability limit nationwide data collection. Research underfunding historically led Brazilian researchers to rely on voluntary efforts for data collection. The IMPACTO-MR platform can overcome these barriers by providing funding for data collection in all participant ICUs (guaranteed until 2023), along with multidisciplinary site staff training (nurses, research assistants, doctors, laboratory staff, and infection control staff) and a single data collection system focused on critical variables, which can also be used for benchmarking and performance evaluation.

For the first time, Brazilian ICUs have a nationwide representative database allowing for better generalization of results and introduction of platform trials. Furthermore, the system used for data collection is a commercial system widely used for quality improvement and benchmarking. This was an advantage for participant ICUs as data entered into the system are used not only for clinical research but also for management and quality improvement. The direct leadership of prominent research institutions helps guide the database purpose to relevant research prospects.

A gap between clinical practice and clinical research has been acknowledged for a long time. The problem occurs in two ways: the uptake of research evidence into practice, the central aim of evidence-based medicine, is faulty and lengthy. Conversely, the aspiration of learning and generating systematic knowledge from clinical practice is rarely achieved and is far from reality. Research is usually a costly, complex, and bureaucratic endeavor conducted by supplementary individuals, many of whom are not directly involved with patient care. Most studies are stand-alone initiatives with specific databases, which are discontinued after the study conclusion. Therefore, how the research conclusions are incorporated into the clinical practice of even the participating centers is lost. Solutions to overcome this problem are needed. A platform with a continuous collection of routine data of all patients should facilitate embedding multiple observational studies and trials into practice - the care of every patient should generate knowledge. Conversely, the implementation of newly generated evidence from studies conducted on the platform can be systematically measured. However, the project implementation faced some difficulties. First, one of the advantages of the IMPACTO-MR platform, its nationwide representativeness, imposed logistical challenges for implementation and staff training. Second, the lack of a centralized process for IRB approval for observational trials in Brazil led to some disparities in the regulatory phase. One site center demanded obtaining informed consent for all patients admitted to the ICU. Third, despite training and funding, continuous data input for all ICU admissions is a monumental task, implying variability in the data collected in each participant ICU, demanding extra effort directed to data management (curation). Finally, the COVID-19 pandemic, which overwhelmed health care systems throughout the world, led to interruptions in data collection for some ICUs, with some units abandoning the platform.

## CONCLUSION

The IMPACTO-MR platform is a Brazilian nationwide intensive care unit clinical database focused on research on the impact of health care-associated infections due to multidrug-resistant bacteria. With more than 50 intensive care units and more than 70,000 patients included, the platform provides data for individual intensive care unit development and research and multicenter observational and prospective trials.
